# Impaired Glucose Metabolism in Young Offspring of Female Rats with Hypothyroidism

**DOI:** 10.1155/2019/4713906

**Published:** 2019-02-21

**Authors:** Zhoujun Liu, Yu Chen, Guofang Chen, Xiaodong Mao, Xiao Wei, Xingjia Li, Yijiao Xu, Fan Jiang, Kun Wang, Chao Liu

**Affiliations:** ^1^The First College of Clinical Medicine, Nanjing University of Chinese Medicine, Nanjing, China; ^2^Department of Endocrinology, Affiliated Hospital of Integrated Traditional Chinese and Western Medicine, Nanjing University of Chinese Medicine, Nanjing, China; ^3^Endocrine Research Center, Affiliated Hospital of Integrated Traditional Chinese and Western Medicine, Nanjing University of Chinese Medicine, Nanjing, China; ^4^The Third College of Clinical Medicine, Nanjing University of Chinese Medicine, Nanjing, China; ^5^The Affiliated Jiangning Hospital of Nanjing Medical University, Nanjing, Jiangsu, China

## Abstract

**Purpose:**

Because thyroid hormones from the maternal thyroid glands are known to influence the growth, development, and metabolic functioning of offspring, we used a rat model to preliminarily investigate the effects of maternal hypothyroidism on glucose metabolism, pancreas cell proliferation, and insulin production in young male offspring and the possible underlying mechanisms.

**Methods:**

Female rats were divided into a maternal hypothyroidism (MH) group, which received water containing 0.02% 6-propyl-2-thiouracil before and during pregnancy to induce hypothyroidism, and a control group which consumed tap water.

**Results:**

Our results showed that there were no differences of islets structure between the offspring from the two groups, but glucose metabolism was impaired with higher plasma glucose concentrations at 0 and 15 min in the OGTT in 8-week-old offspring of the MH group. From birth to 8 weeks, pancreatic TR*β*1 and TR*β*2 mRNA level declined significantly in MH offspring, accompanied by decreased Ki67 and insulin mRNA expression.

**Conclusions:**

Maternal hypothyroidism results in impaired pancreatic insulin synthesis and pancreatic cell proliferation in neonatal offspring and subsequent glucose intolerance in young offspring, which may be related to TR*β* gene downregulation in the pancreas.

## 1. Introduction

Since the theory of fetal origins of adult disease (FOAD) was posed by Barker [1], much effort has been directed at understanding the relationship between maternal hypothyroidism and adult degenerative and metabolic diseases. Research has linked asymmetrical intrauterine growth retardation (IUGR) as well as decreased muscle mass to maternal hypothyroidism [2], and studies have also reported deficiencies in the development of the nervous system, appendicular skeleton, skeletal muscle [3], skin, lungs, and pancreas [4, 5] of offspring. Recently, the relationship between maternal hypothyroidism and glucose metabolic abnormalities in adult offspring has been increasingly emphasized, with research in rats showing that maternal hypothyroidism causes IUGR and diminished insulin secretion capacity in adult offspring [6]. But few findings had focused on the glucose metabolism for the young offspring of female rats with hypothyroidism.

It is known that thyroid hormone is an important factor to maintain the proliferative and secretory ability of pancreatic *β* cells. So the maternal level of thyroid hormone is crucial for the development of *β* cells in offspring. Evidence showed that most effects of thyroid hormone are mediated by nuclear thyroid hormone receptors (TRs), including TR*α* and TR*β*. They act as transcription factors [7, 8] and are abundantly expressed in pancreatic islets [9]. Three main isoforms, TR*α*1, TR*β*1, and TR*β*2, are expressed in the whole pancreas during development [10], and these isoforms regulate gene expression in a similar ligand-dependent manner [11]. Within islets, TR*α*1 is mainly expressed in the nucleus of *α* cells where it can modulate glucagon gene expression [12, 13], and TR*β* is present in the *β* cells where it can bind with thyroid hormone, a key factor in *β* cell proliferation. TR*β* gene knockout mice showed early *β* cell apoptosis and then insulin secretion dysfunction in adulthood [14]. Moreover, upregulation of TR*β* leads to increased insulin secretion [15]. Thus, the expression level of TR*β* strongly affected the ability of thyroid hormones to exert their normal biological activities.

Therefore, to simulate a more real state of hypothyroidism in pregnancy, the present study established the model of hypothyroidism in female rats first and then mate them with normal male rats to preliminarily explore the underlying mechanisms that maternal hypothyroidism may affect metabolic function of young male offspring.

## 2. Materials and Methods

### 2.1. Animals and Treatment

Virgin female Sprague-Dawley (SD) rats were purchased from Experimental Animal Center, Nantong University (Nantong, China), and kept in the Affiliated Hospital of Integrated Traditional Chinese and Western Medicine animal facility. They were exposed to standard conditions (temperature 22 ± 2°C; relative humidity 24 ± 6%; 12 h light/dark cycle) with free access to food and water. Once these female rats reached a weight of 180–220 g, they were randomly divided into the control group (*n* = 30) and the MH group (*n* = 55). The maternal hypothyroidism (MH) group received 0.02% (200 ppm) 6-propyl-2-thiouracil (PTU) (Sigma-Aldrich, USA) in drinking water to induce hypothyroidism, whereas the control group consumed tap water [16, 17]. Two weeks later, once hypothyroidism was established by PTU treatment in the MH group, the female rats of both groups were placed in cages with male rats overnight at a ratio of one female to one male. Observation of a vaginal plug indicated pregnancy, and 21 rats in the control group and 20 rats in MH group were pregnant. Fifteen rats from both groups that were chosen for this study are pregnant at the same period. In the MH group, PTU treatment was continued throughout pregnancy and discontinued after delivery. Litter size, offspring mortality rates, gestational length, and maternal weight gain (%) were recorded for each female rat. The protocols for all procedures involving animals were approved by the Animal Care and Use Committee of Affiliated Hospital of Integrated Traditional Chinese and Western Medicine.

### 2.2. Ethics Approval of the Study Protocol

All animal protocols for this study conformed to the Guide for the Care and Use of Laboratory Animals published by the US National Institutes of Health. All experimental procedures were approved by the Animal Care and Use Committee of Nanjing University of Traditional Chinese Medicine (Nanjing, China).

### 2.3. Oral Glucose Tolerance Test (OGTT)

Male offspring from both groups were subjected to an OGTT at 4 and 8 weeks of age. After fasting for 16 h overnight, the rats were weighed for calculation of the glucose dosage. First, blood samples were taken from cutting tails to determine the blood glucose at time zero. Then, all rats received 2 g/kg glucose via oral lavage. At 15, 30, 60, and 120 min after glucose loading, the blood glucose was measured by an automatic glucometer (Roche, Germany).

### 2.4. Serum Thyroxine and Thyroid-Stimulating Hormone (TSH) Assay

After delivery, neonates were anesthetized by intraperitoneal injection of sodium pentobarbital (30 mg/kg), and blood was drawn via apical blood extraction, and every blood sample was pooled from 6 to 8 pups. Blood samples of mothers and the young offspring were drawn from the orbit fossa. Blood (about 500 *μ*l) was collected in microcentrifuge tubes and centrifuged at 3000 rpm for 15 min, and about 200 *μ*l serum could be obtained after centrifugation. Serum thyroid hormone levels (T4 and TSH) were measured using the MILLIPLEX MAP Rat Thyroid Hormone Panel kit (Millipore, USA).

### 2.5. Histopathological Evaluation

The pancreas segment of the rats in each group was fixed in 4% paraformaldehyde and dehydrated through a grade ethanol series, soaked in xylene, and embedded in paraffin. The segment was cut into sections with 5 *μ*m thickness. The histopathological examinations of the sections were performed with hematoxylin and eosin (H&E) staining and evaluated by the light microscope.

### 2.6. Localization of Ki67 and Insulin via Immunolabeling

The pancreatic tissue section was deparaffinized, rehydrated, and fixed in 4% paraformaldehyde. Then, the tissues were permeated with 0.3% Triton X-100 for 5 min and incubated with 5% BSA for 30 min at room temperature. The anti-insulin (1 : 200) and anti-Ki67 (1 : 200) antibodies bought from Abcam Inc. (England) were incubated at 4°C overnight, followed by incubation with secondary antibodies (Invitrogen, Carlsbad, CA) for 30 min. Nuclei were stained with DAPI. Ki67 and insulin expression was detected using a fluorescence microscope (Olympus ix71, Japan).

### 2.7. Quantitative Real-Time PCR (qRT-PCR) for Determining mRNA Expression of Ki67, Insulin, and TR*β*

Total RNA was extracted from the pancreas of male offspring using TRIzol reagent (Thermo Fisher Scientific, USA). The purity of RNA was determined using the Synergy H1 Hybrid Reader Take3 Plate (BioTek, USA). Primers were dissolved in water to the recommended concentrations according to Generay Data Sheets (10 nmol/ml). The ReverTra Ace qPCR RT kit (Toyobo, Japan) was used for reverse transcription. qRT-PCR was performed with Quant Studio Dx System (Life Technology, USA) using the SYBR Select Master Mix kit (Applied Biosystems, USA). The PCR cycling were 95°C for 1 min and then 40 cycles of 95°C for 15 sec, 55°C for 15 sec, and 72°C for 45 sec. Each sample was run in triplicates and normalized to GAPDH. The relative quantification values were determined using the 2^−ΔΔCt^ method. Sequences of primers for mRNA were shown in Table 1.

### 2.8. Statistical Analysis

Data are expressed as mean ± standard error of the mean (SEM). Student's *t*-test was used to compare data from the two groups, and differences with a *p* value of <0.05 were considered to be statistically significant. GraphPad Prism software (version 5) was used for all statistical analysis.

## 3. Results

### 3.1. Thyroid Function of Mothers and Offspring

After treatment with PTU for 14 days, the female rats in the MH group had a higher TSH level and lower T4 level than female rats in the control group (both *p* < 0.001). In our present study, the maternal hypothyroidism model has been established before pregnancy. And the state of maternal hypothyroidism lasted to the time of delivery, demonstrated by elevated serum TSH and declined T4 level. In addition, the serum TSH level of neonates born in the MH group also was significantly higher than that in the control group (*p* < 0.05). However, no differences were observed in the serum TSH and T4 concentrations between young male offspring from mothers in the MH and control groups (Table 2).

### 3.2. Pregnancy Outcome and Fetal Growth Rate

**Figure 1:**
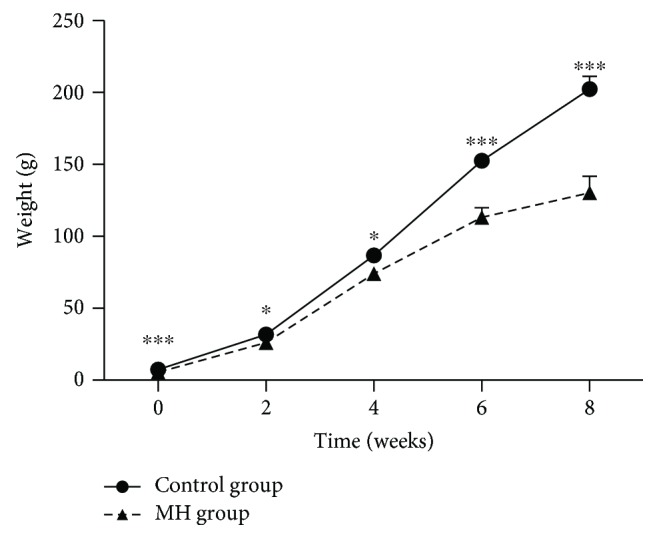
Body weight measurement of male offspring. Data are mean ± SEM. ^∗^*p* < 0.05, ^∗∗^*p* < 0.01, and ^∗∗∗^*p* < 0.001; *n* = 20.

The litter size did not differ between the MH and control groups (*n* = 15 female rats/group). The total numbers of pups born from the control and MH group were 184 and 150, respectively. The mortality rate of offspring of the MH group was 6.7% (10/150), which has no statistical difference with that of control group (4.3%) (8/184) (Table 3). And there was also no statistical difference in gestational length. The maternal weight gain in the MH group was significantly lower than that in the control group (Table 3). Consistent with the lower maternal weight gain in the MH group, neonates born from mothers in the MH group had a significantly lower birth weight than those born from mothers in the control group (Table 3), and the offspring of the MH group also showed significantly lower weight gain by the age of 8 weeks compared to the offspring of the control group (Figure 1). In addition, the random blood glucose level at birth in the offspring of the MH group was significantly higher than that in the offspring of the control group (Table 3).

### 3.3. OGTT Performance

**Figure 2:**
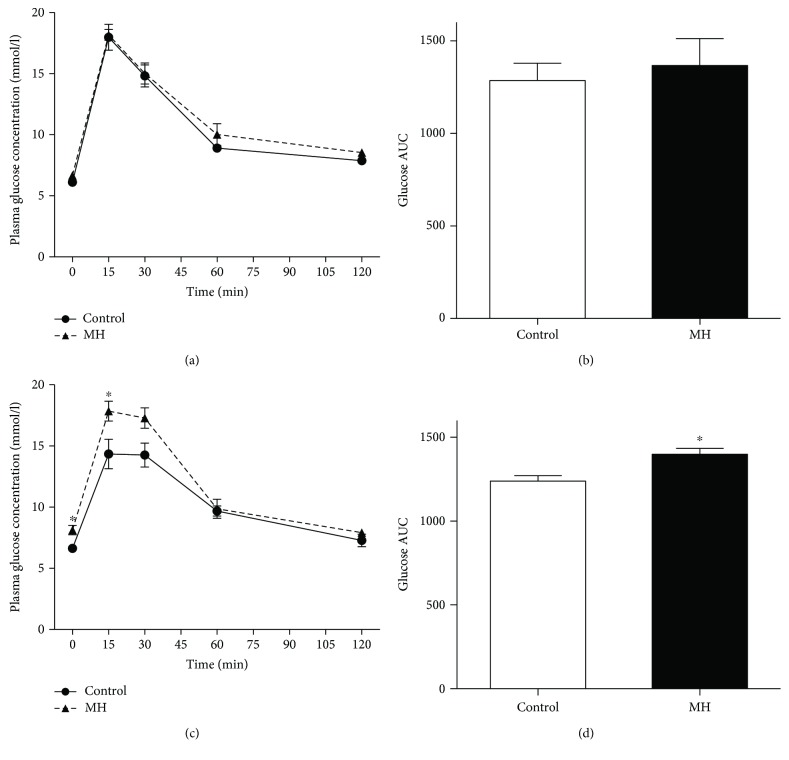
Comparison of changes in plasma glucose concentration during OGTT in the control and MH groups. Plasma glucose concentration following OGTT at 4 weeks old (a) and 8 weeks old (c) of the two groups. The glucose AUC for male offspring in both groups at ages 4 weeks (b) and 8 weeks (d). Data are mean ± SEM. ^∗^*p* < 0.05 between groups.

The plasma glucose levels measured by OGTT in the offspring from mothers in the MH and control groups are shown in Figure 2. In the 4-week-old rats, no differences were observed in the plasma glucose levels at any measurement time (Figure 2(a)) or in the area under the glucose curve (AUC; Figure 2(b)) between the offspring of the MH and control groups. However, in the 8-week-old rats, the results of OGTT showed that plasma glucose levels under fasting conditions (0 min) were higher in offspring of the MH group than that in offspring of the control group (8.13 ± 0.38 mmol/l vs 6.63 ± 0.19 mmol/l, *p* < 0.05, Figure 2(c)). Fifteen minutes after glucose administration, the plasma glucose levels increased to a higher level in the offspring of the MH group compared with those of the control (17.84 ± 0.81 mmol/l vs 14.34 ± 1.21 mmol/l, *p* < 0.05). Quantitatively, the AUC was significantly greater for the offspring of the MH group than that for the offspring of the control group (1399 ± 35.46 mmol l^−1^ (120 min)^−1^ vs 1239 ± 31.7 mmol l^−1^ (120 min)^−1^, *p* = 0.01; Figure 2(d)).

### 3.4. Histological Evaluation

**Figure 3:**
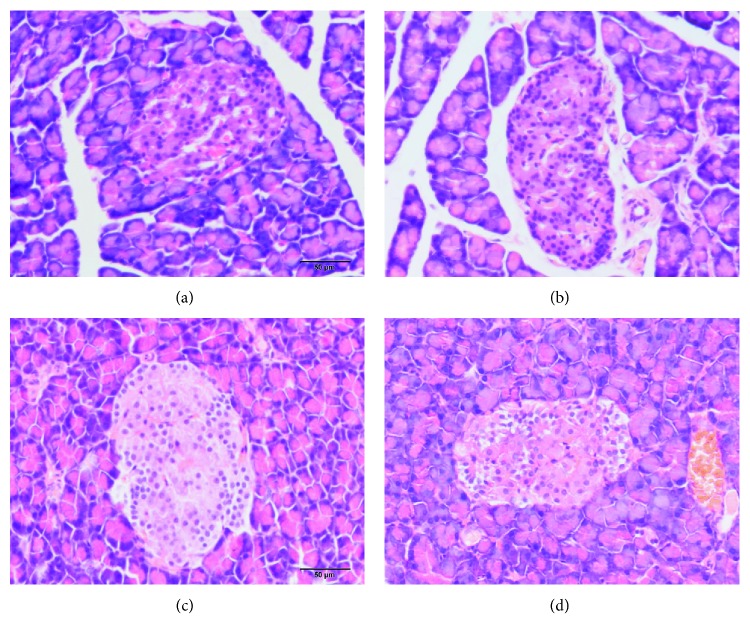
(a, b) The H&E staining of the pancreas (400x) in male offspring of the control (a) and MH (b) groups at 4 weeks old. (c, d) The H&E staining of the pancreas (400x) in male offspring of the control (c) and MH (d) groups at 8 weeks old.

The pancreatic sections in the male offspring from the MH and control groups stained with H&E were shown in Figure 3. The results showed that the islet morphology in the offspring from the MH group did not differ from the offspring of the control group at 4 weeks old and 8 weeks old.

### 3.5. Detection of TR*β* mRNA Expression in the Pancreas

**Figure 4:**
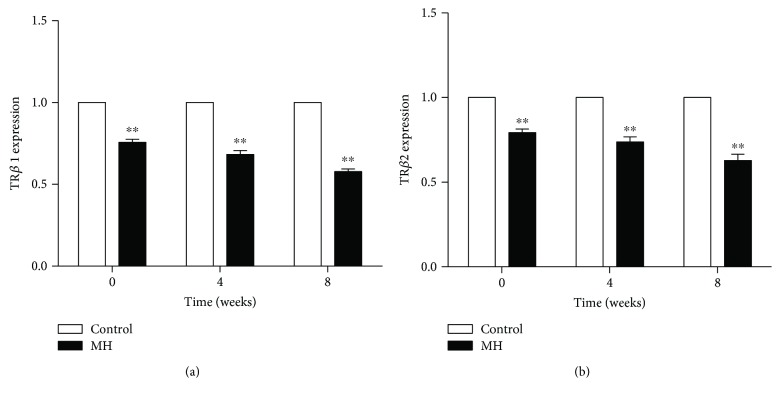
Comparison of pancreatic TR*β*1 mRNA (a) and TR*β*2 mRNA (b) in the control and MH male offspring. Data are expressed as mean ± SEM, ^∗∗^*p* < 0.01.

The TR*β*1 (Figure 4(a)) and TR*β*2 (Figure 4(b)) mRNA levels in the offspring of the MH group were significantly lower than those in the offspring of the control group at birth, at 4 weeks of age, and at 8 weeks of age.

### 3.6. Detection of Insulin Synthesis and Cell Proliferation in the Pancreas

**Figure 5:**
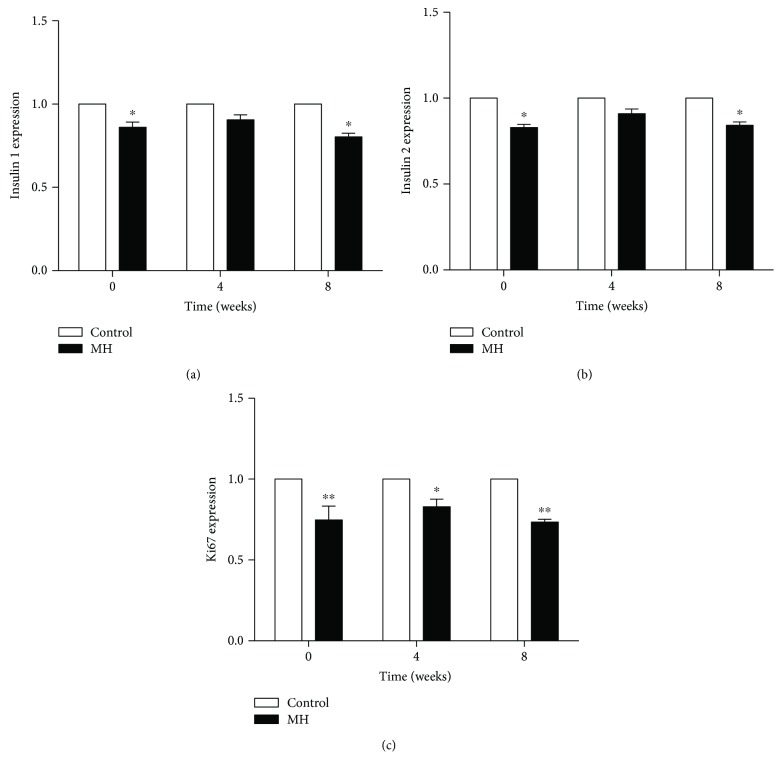
Comparison of pancreatic insulin and Ki67 mRNA expression in the control and MH male offspring from 0 to 8 weeks old. Data are expressed as the mean ± SEM (^∗^*p* < 0.05 and ^∗∗^*p* < 0.01 compared with control).

**Figure 6:**
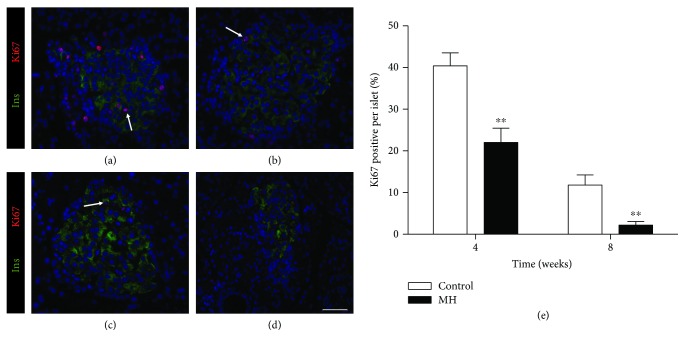
(a, b) Anti-Ki67 and anti-insulin immunostaining of islet cells in the control (a) and MH (b) male offspring rats at 4 weeks old. (c, d) Anti-Ki67 and anti-insulin immunostaining of islet cells in the control (c) and MH (d) offspring male rats at 8 weeks old. Arrows indicate immunopositive nuclei. Bar = 50 *μ*m. (e) Percentage of proliferative cells per islet considering overall randomized selected islets of the control and MH male offspring. Asterisks indicate significant differences between treatments. ^∗^*p* < 0.05.

The insulin synthesis and cell proliferation of pancreatic *β* cells were detected by the expression of insulin and Ki67 via PCR (Figure 5) and immunolabeling (Figure 6). Our results showed that the mRNA levels of insulin 1 (Figure 5(a)) and insulin 2 (Figure 5(b)) in the offspring of the MH group were significantly lower than those in the offspring of the control group at birth and 8 weeks of age. Meanwhile, we measured the mRNA expression of Ki67, a marker of cell proliferation. The gene expression in the pancreas of the MH group decreased compared with control from birth to 8 weeks old (*p* < 0.01, Figure 5(c)).

## 4. Discussion

Our present study showed that hypothyroidism leads to reduced maternal weight gain during the gestational period and a prolonged gestational period. Thyroid hormones are key factors in maintaining the balance between energy expenditure and food intake [18, 19], and food intake by mothers with hypothyroidism was found to be significantly lower than that by control mothers [20], which may be explained by the lower weight gain during pregnancy observed in the MH group in our present study. The gestational period appeared to be extended in the MH group compared with the control group, which is similar to other researches [6, 21].

The potential effects of maternal hypothyroidism on the glucose metabolism in offspring were brought into focus recently. In our present study, PTU treatment increased the TSH level and decreased the T4 level in mothers, accompanied by elevated TSH level in their neonates compared with those consuming tap water, confirming the induction of maternal hypothyroidism. Serum T4 and TSH levels in offspring male rats at young stage did not differ between the MH and control groups, indicating the offspring of the MH group return to a euthyroid state. Previous study from Karbalaei et al. [21] also proved normal thyroid hormone levels of adult offspring rats born form maternal hypothyroidism.

Our results showed that maternal hypothyroidism led to restricted growth of male offspring from birth to the young period. The body weights of the male pups of the MH group at birth and later on were significantly lower than those in the control group (until 8 weeks of age). The litter size in the MH group shows a trend of reduction though without a statistical difference which is in line with the trend presented by Karbalaei et al. [21]. It is well known that thyroid hormones play an important role in the growth, development, and metabolism of essentially all tissues, particularly in the growing fetus [2, 22]. Smaller litter size and low birthweight imply that maternal hypothyroidism could lead to fetal organ development retardation.

The onset of impaired glucose level was observed in male offspring of the MH group from birth which manifested by increased randomized plasma glucose. When the pups grew up to 4 weeks old, the glucose metabolism of the offspring went back to normal performance which is similar to the control group. However, the increased fasting and postprandial plasma glucose as well as plasma glucose AUC responses to OGTT were observed in the young male offspring of the MH group at 8 weeks old. Our results confirmed that maternal hypothyroidism would have adverse effects on glucose metabolism in young offspring. It is noteworthy that the age of offspring from mothers with hypothyroidism presenting abnormal glucose metabolism in our present study is much earlier compared with the findings of other similar studies [6, 23]. To the best of our knowledge, insulin synthesis and secretion, as well as cell proliferation, are essential factors in pancreatic *β* cells to maintain glucose homeostasis. So, we tested the gene expression of insulin and Ki67—a marker of cell proliferation, in the pancreas of male offspring from the two groups. The results showed that insulin 1, insulin 2, and Ki67 mRNA expressions all decreased significantly in the offspring of the MH group from 0 to 8 weeks old, except insulin expression at 4 weeks old. The alterations of their expression were also validated in protein level by immunofluorescent staining. Thus, we speculated that the abnormal glucose homeostasis at these time points may be due to insufficient synthesis of insulin and inhibited proliferation of pancreatic *β* cells.

Large number of researches have been demonstrated that thyroid hormones were crucial regulators to promote *β* cell proliferation and insulin secretion capacity in islets [24]. However, constant low levels of insulin synthesis and cell proliferation in offspring after birth are not likely to be due to transient subclinical hypothyroidism. We thought these results in offspring were caused by the intrauterine hypothyroid environment. It has been reported that the thyroid axis in the human embryo begins to develop at E90 [25, 26]. The thyroid gland develops from the floor of the primitive pharynx and can first be distinguished in the rodent embryo at 9 days post coitum [27]. Therefore, the growth and development of the embryo during the early stage rely on the maternal thyroid hormone. Maternal thyroid hormone was shown to enter the embryo by binding thyroid hormone transporters in the placenta and to affect embryo development [28]. It is classically known that most of the effects of bioactive thyroid hormones are mediated by TRs, and a previous study showed that early fetal brain development is regulated by maternal thyroid hormone levels mediated by TRs [29]. Another study found that the risk of heart failure in postnatal life is related to the maternal thyroid function regulated by TR*α* in the cardiac cells of the embryo [30]. In our present experiment, the pancreatic TR*β*1 and TR*β*2 expressions of offspring from the MH group maintained at a lower level from birth to young compared with those in the offspring of the control group. Based on these research results, it can be concluded that the development, growth, and normal regeneration of the offspring's pancreas may be affected by maternal thyroid function through modulated by TRs. Combined with our previous findings, several genes presented adaptive expression with proliferation of pancreatic islets during pregnancy in rats [31]. We speculate that permanent alteration of TR*β* expression may be a genetic adaption to intrauterine hypothyroidism that could lead to islet cell dysplasia.

In conclusion, maternal hypothyroidism can slow intrauterine growth of a fetus and cause low expression of TR*β* in the pancreas, both of which result in deficiencies in pancreas' cell proliferation and insulin synthesis. These effects can lead to long-term alteration of glucose metabolism in offspring, and we are proceeding with in vitro experiments to verify whether thyroid hormone directly promotes proliferation of *β* cells via signaling involving TR*β*. Moreover, whether hypothyroidism in the early stage of pregnancy has a decisive impact on these results deserves further research.

## Figures and Tables

**Table 1:** Primers used for RT-PCR.

Target gene	Primer sequence (5′-3′)		Size (bp)
	Forward	Reverse	
TR*β*1	TCATCCAGGTTGAAAGACGACAG	GCTATGACCCAGACAGCGAGACT	142
TR*β*2	TGTCAGCCGCACGCCTACAAGTC	TAGTGCCGCCTCCAGGAGCGATAT	186
Insulin 1	CCAAGTCCCGTCGTGAAGT	GGTGCAGCACTGATCCACAA	137
Insulin 2	GTGACCAGCTACAGTCGGAA	GCTTCCACCAAGTGAGAACCA	162
Ki67	GGGTTTCCAGACACCAGACC	GGGTTCTAACTGGTCTTCCTGG	101
GAPDH	GCAAGGATACTGAGAGCAAGAGAG	TCCTGTTGTTATGGGGTCTGG	118

**Table 2:** Serum T4 and TSH concentrations of mothers and male offspring.

Hormone	Mothers				Offspring					
	MH model establishment		At delivery		At birth		4 weeks		8 weeks	
	Control (*n* = 15)	MH (*n* = 15)	Control (*n* = 15)	MH (*n* = 15)	Control (*n* = 6)	MH (*n* = 6)	Control (*n* = 6)	MH (*n* = 6)	Control (*n* = 6)	MH (*n* = 6)
TSH (ng/ml)	2.11 ± 0.32	5.63±0.26^∗∗∗^	1.33 ± 0.29	4.85±0.06^∗∗∗^	2.37 ± 0.28	3.08 ± 0.05^∗^	1.98 ± 0.07	1.95 ± 0.18	1.56 ± 0.11	1.75 ± 0.08
Thyroxine (T4, *μ*g/dl)	4.40 ± 0.40	0.97±0.12^∗∗∗^	4.10 ± 0.24	1.17±0.14^∗∗∗^	0.37 ± 0.06	0.33 ± 0.02	2.75 ± 0.14	2.36 ± 0.38	3.63 ± 0.46	4.73 ± 0.83

Values are mean ± SEM. ^∗^*p* < 0.05, ^∗∗∗^*p* < 0.001. MH: maternal hypothyroidism.

**Table 3:** Comparison of pregnancy and neonatal characteristics.

Parameter	Control group	MH group
Pregnancy rate (%)	70	36.36^∗^
Gestational length (days)	22.8 ± 0.8	23.2 ± 0.6
Maternal weight gain during pregnancy (g)	154.8 ± 9.7	118.5 ± 9.5^∗^
Litter size (*n*)	12.3 ± 1.6	10.0 ± 2.0
Birth weight (g)^a^	7.3 ± 0.2	5.6±0.1^∗∗∗^
Offspring mortality rate (%)	4.3%	6.7%
Random glucose level in offspring at birth (mmol/l)	6.47 ± 0.10	7.05 ± 0.21^∗^

^a^The average weight of pups in each litter was computed and reported as a single point by weighing all pups in a litter. Data were obtained for all litters in each group, and the mean values were reported as the group mean in the table. Data are mean ± SEM. ^∗^*p* < 0.05 and ^∗∗∗^*p* < 0.001 compared with the control group.

## Data Availability

The data used to support the findings of this study are available from the corresponding author upon request.
